# Supplementary material for: Chia Seed (Salvia hispanica) Attenuates Chemically Induced Lung Carcinomas in Rats through Suppression of Proliferation and Angiogenesis
Source: Pharmaceuticals (Basel). 2024 Aug 27;17(9):1129. doi: 10.3390/ph17091129 (PMC11435337; doi:10.3390/ph17091129)
Supplement: Supplementary file 1 [file pharmaceuticals-17-01129-s001.zip › pharmaceuticals-3127019-supplementary.pdf]

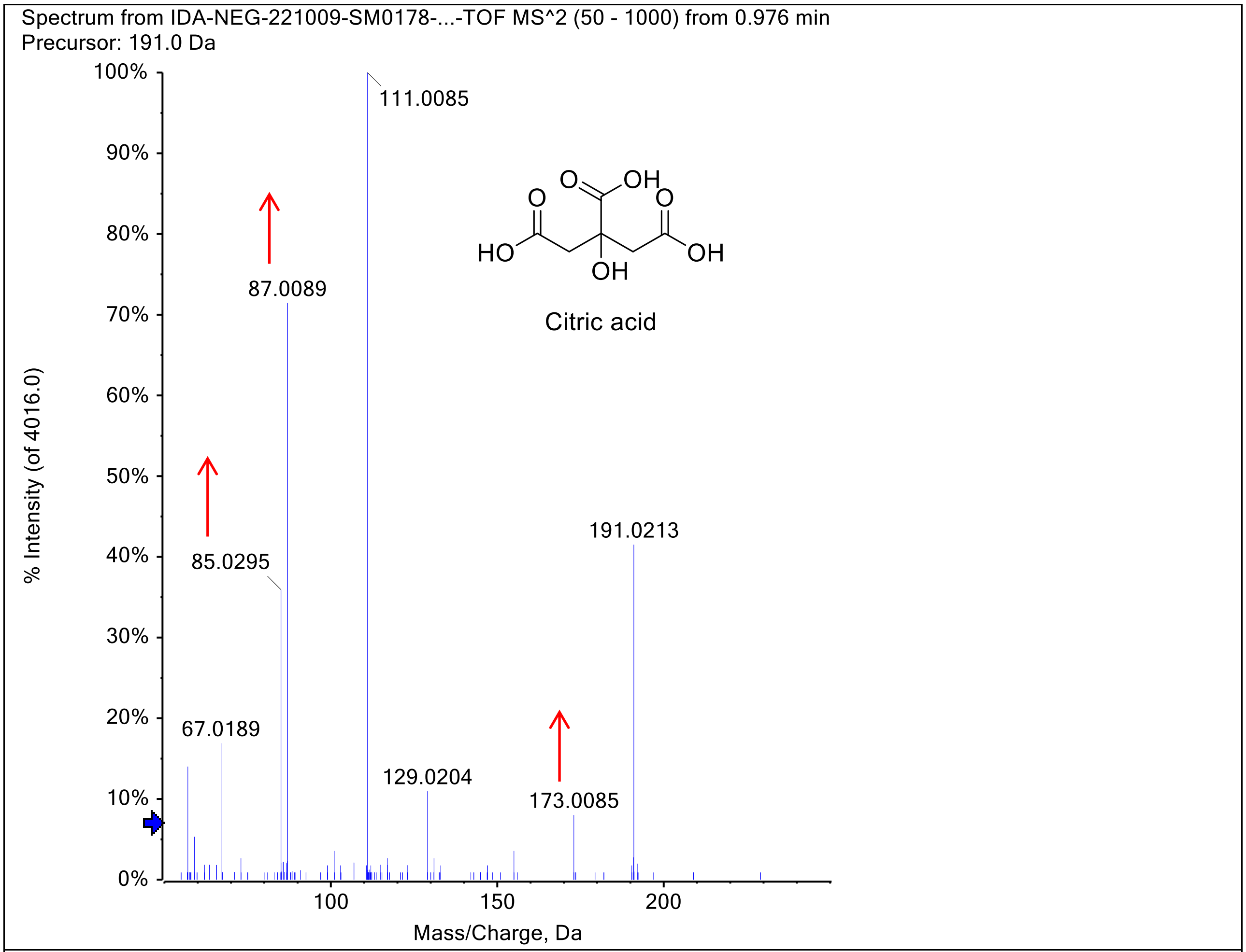

Figure S1. Citric acid

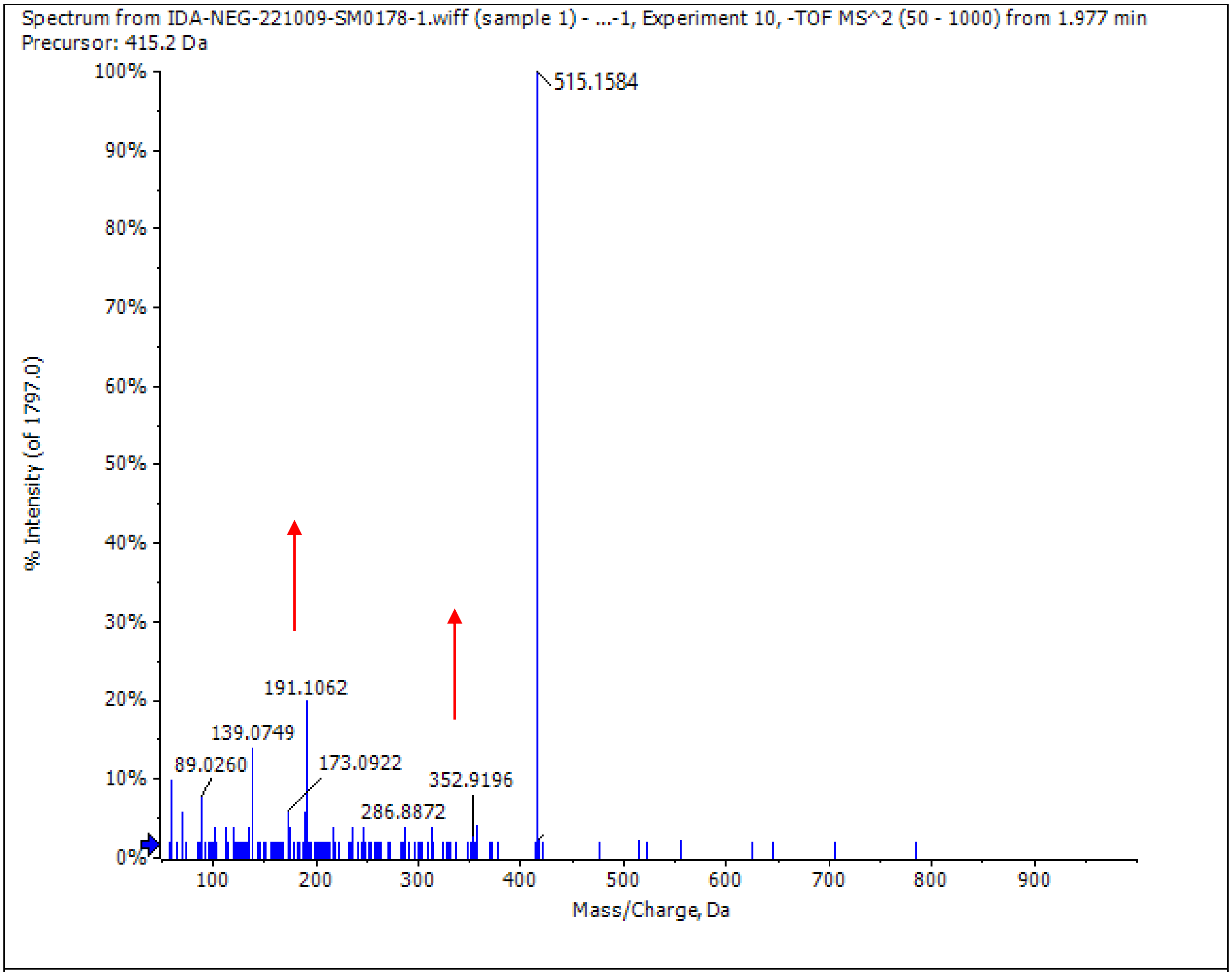

Figure S2. Quinic acid di hexoside

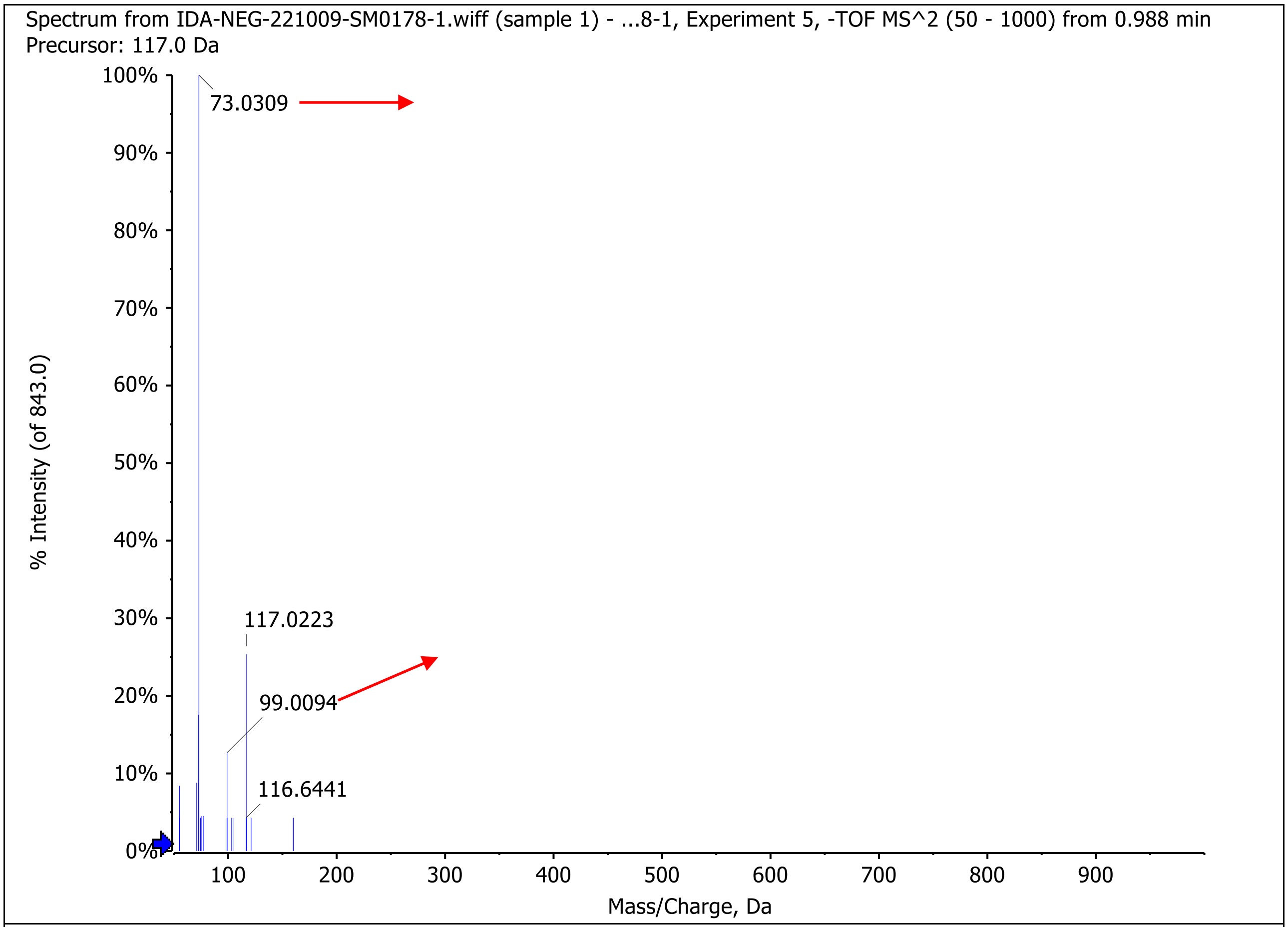

Figure S3. Succinic acid

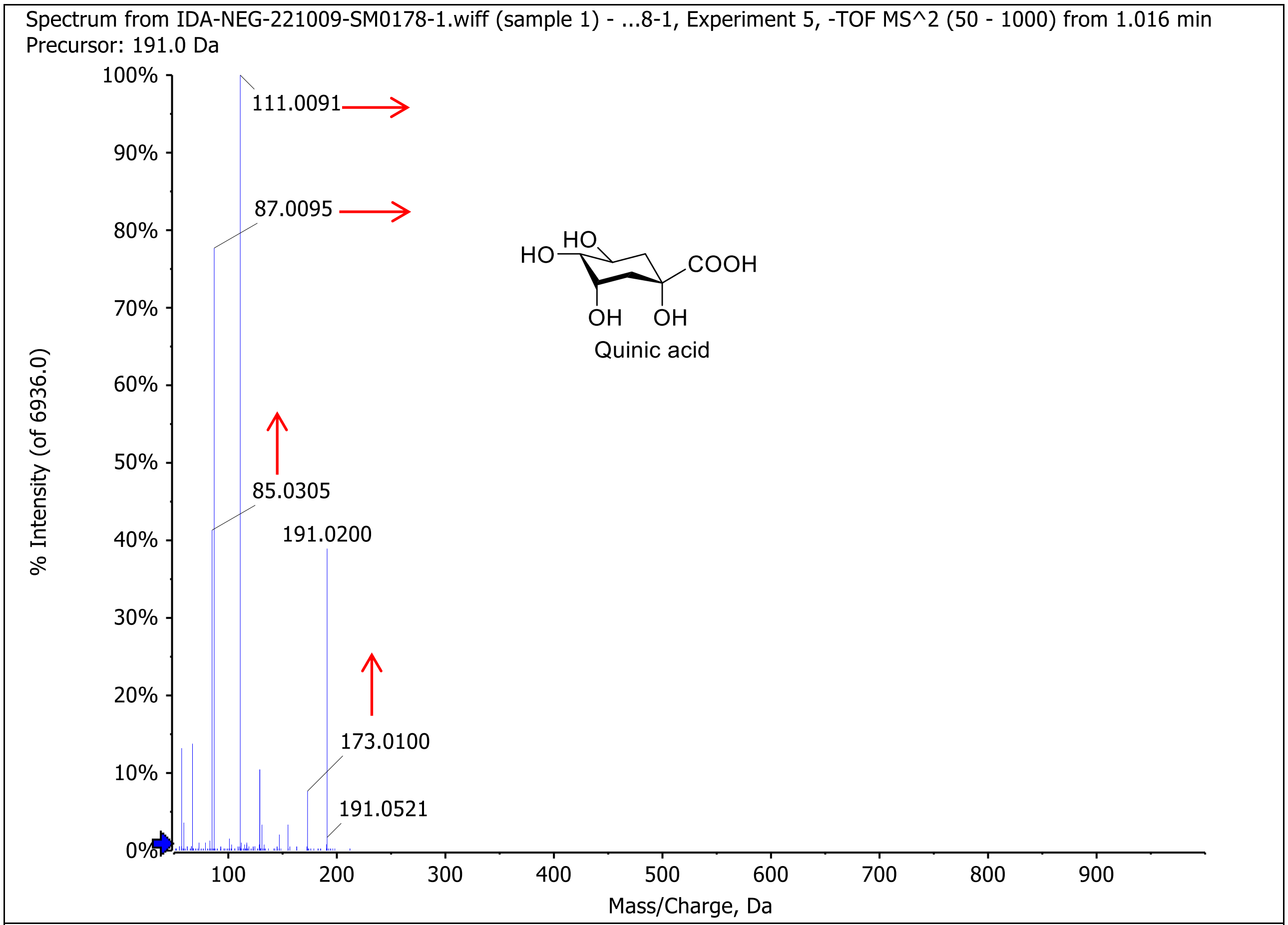

Figure S4. Quinic acid

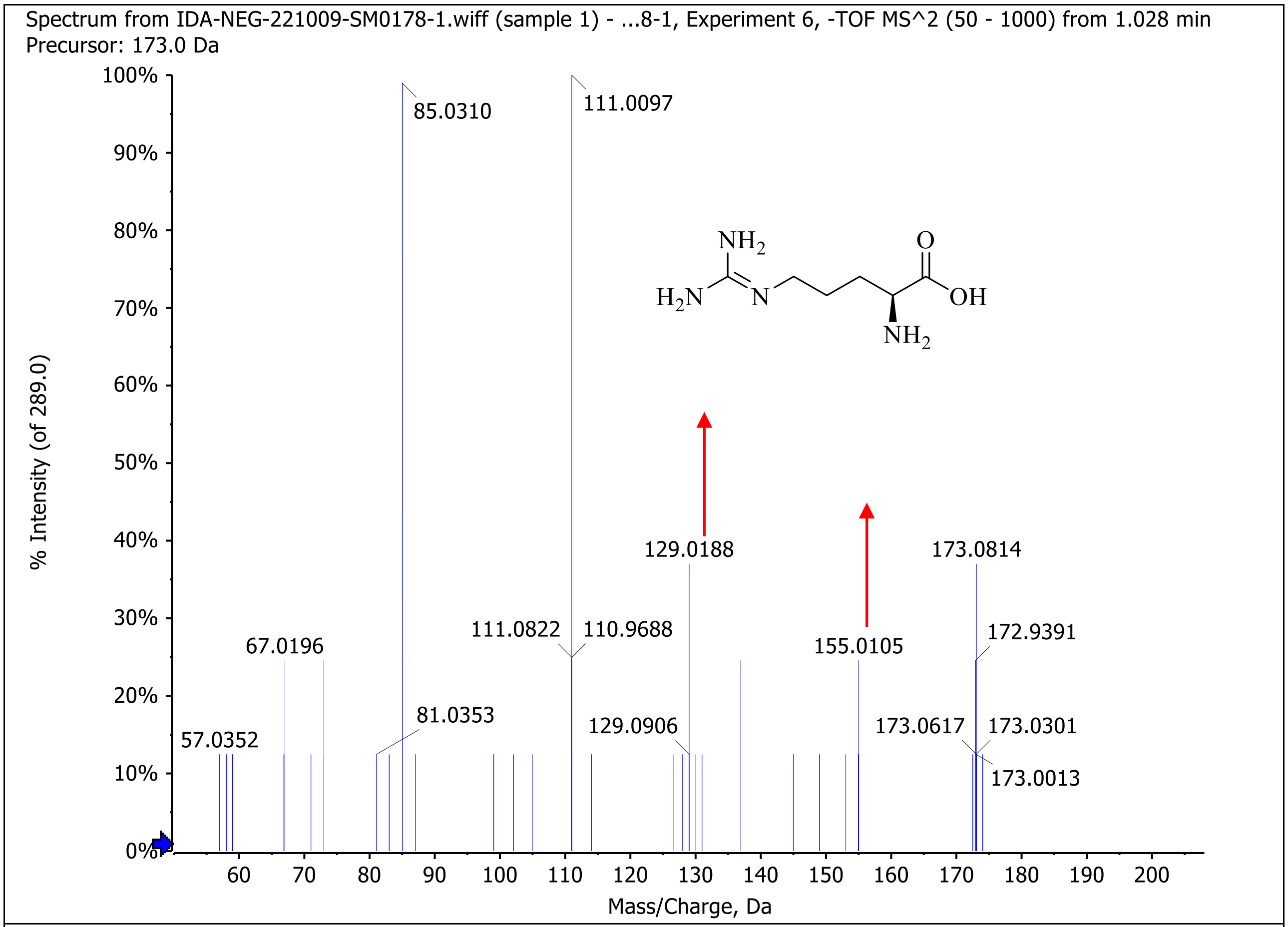

Figure S5. L-Arginine (L-alpha-amino acids) C6H14N4O2

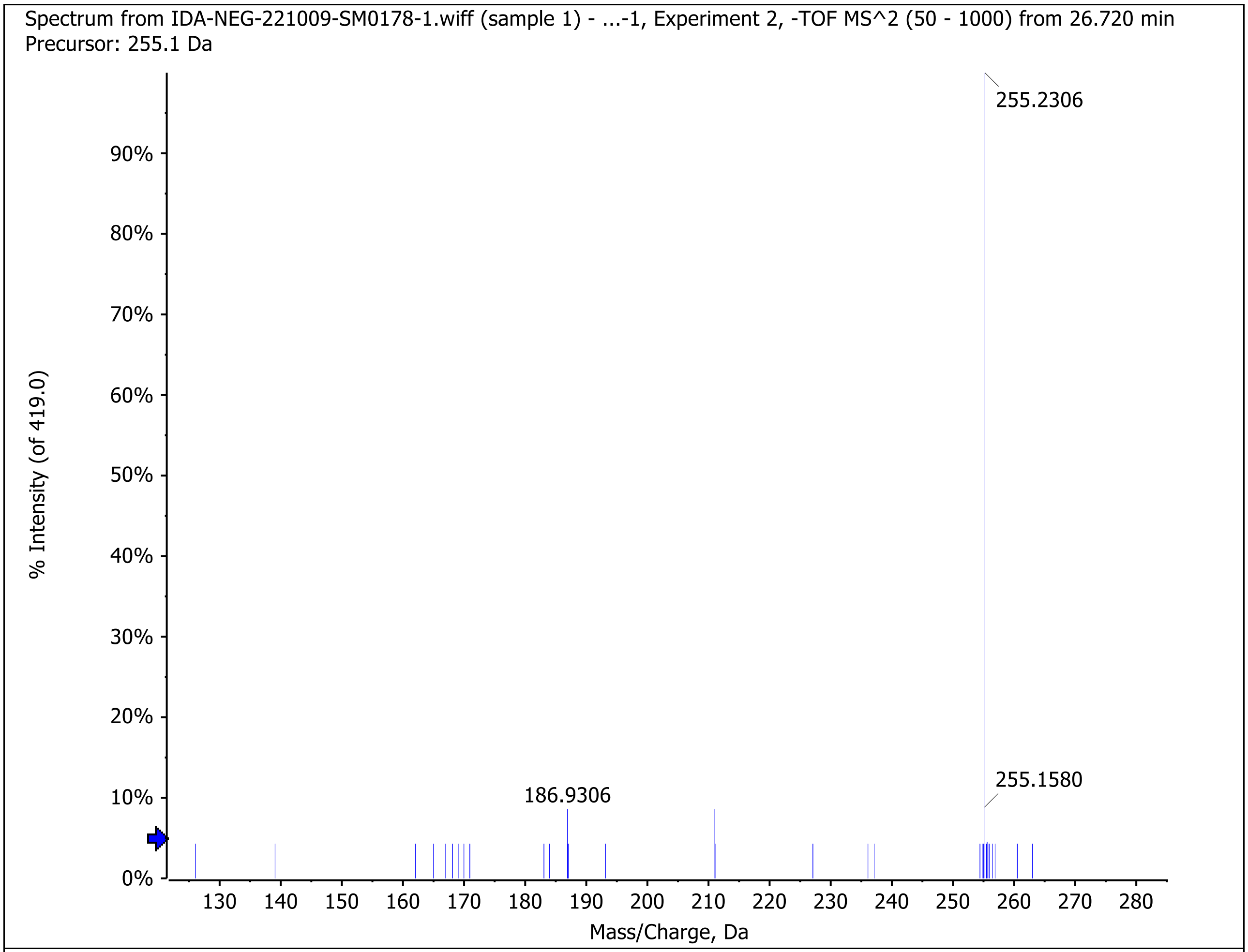

Figure S6. Palmitic acid

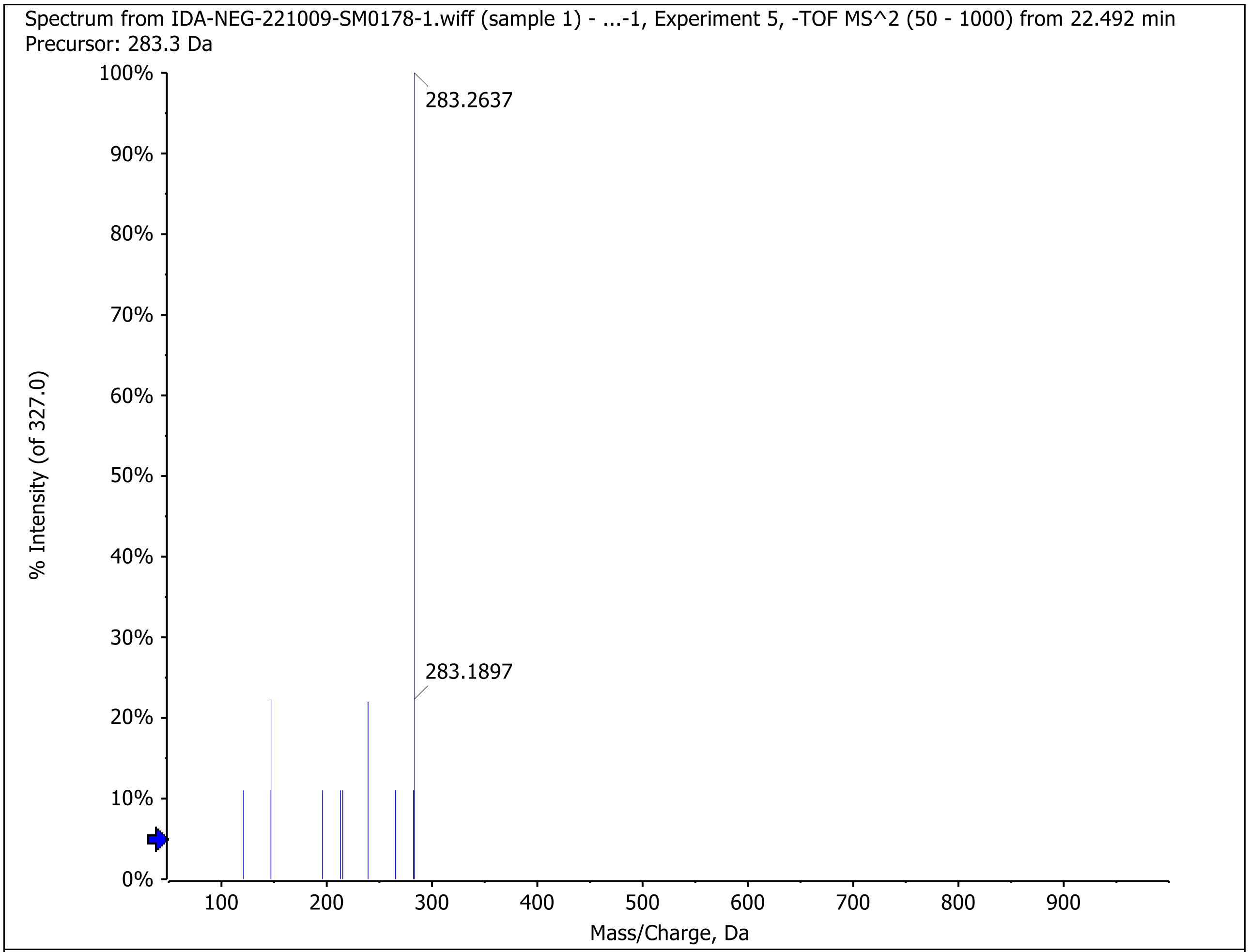

Figure S7. Stearic acid

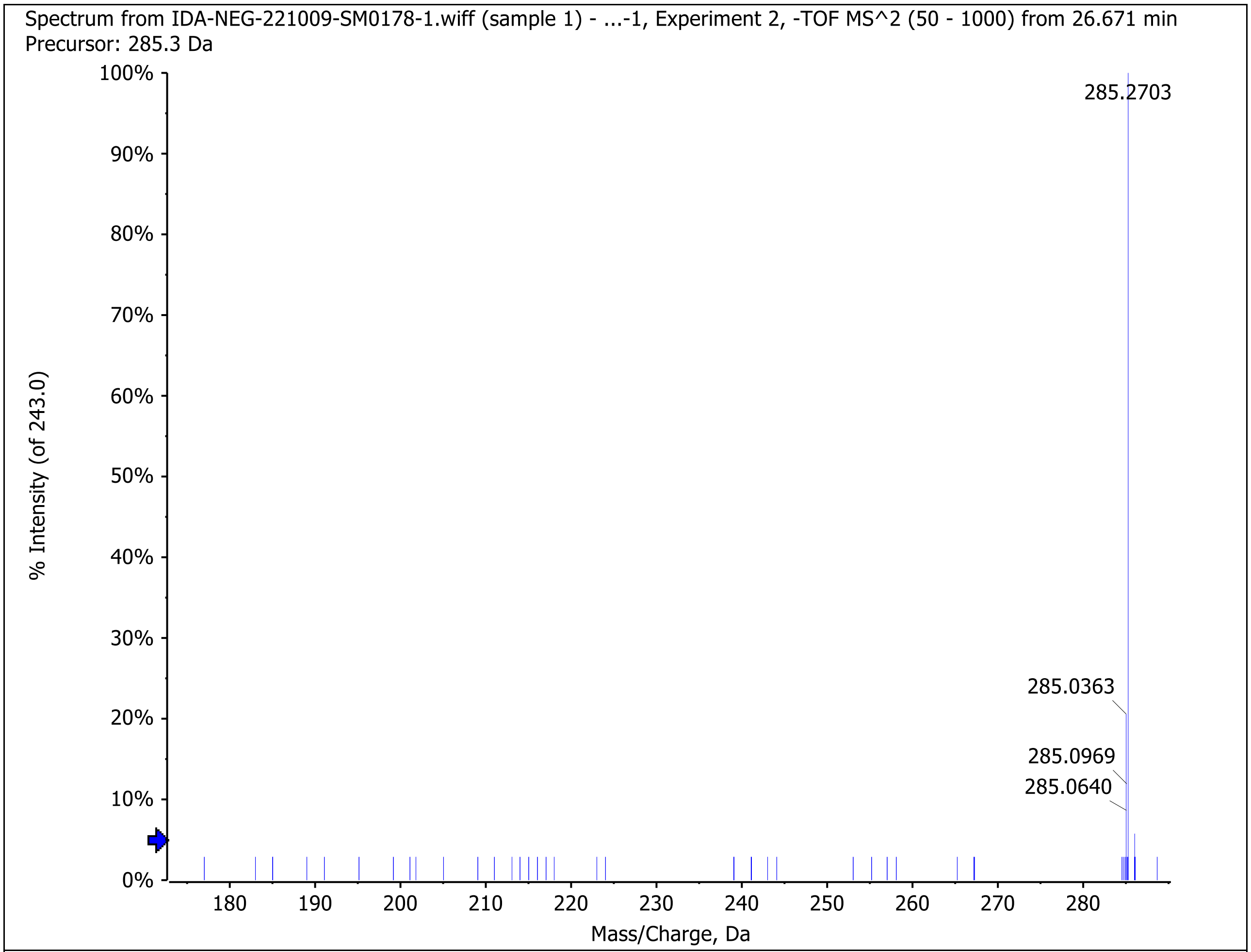

Figure S8. Hydroxy-Oxohexadecanoic acid

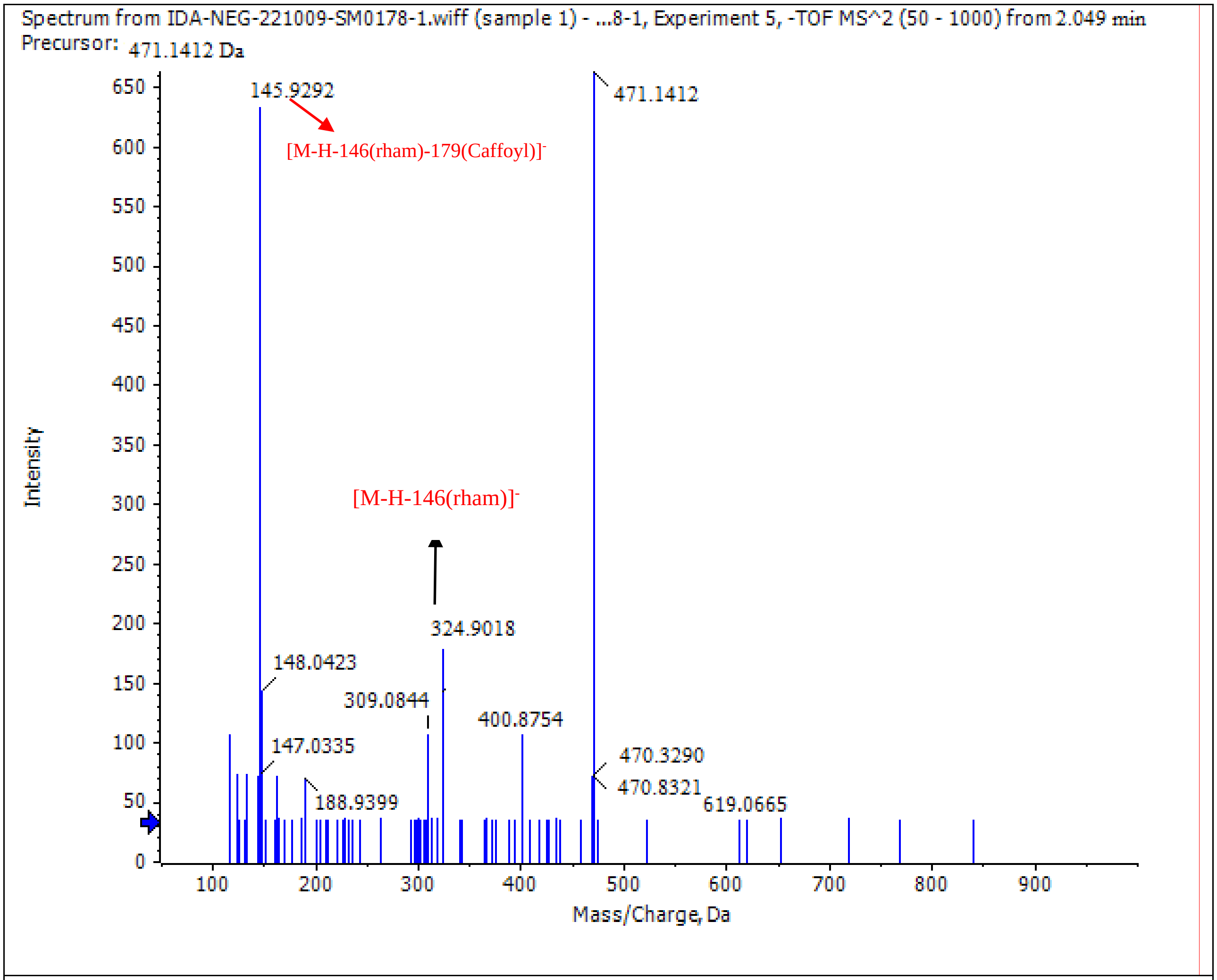

Figure S9. Caffeic acid di rhamnoside

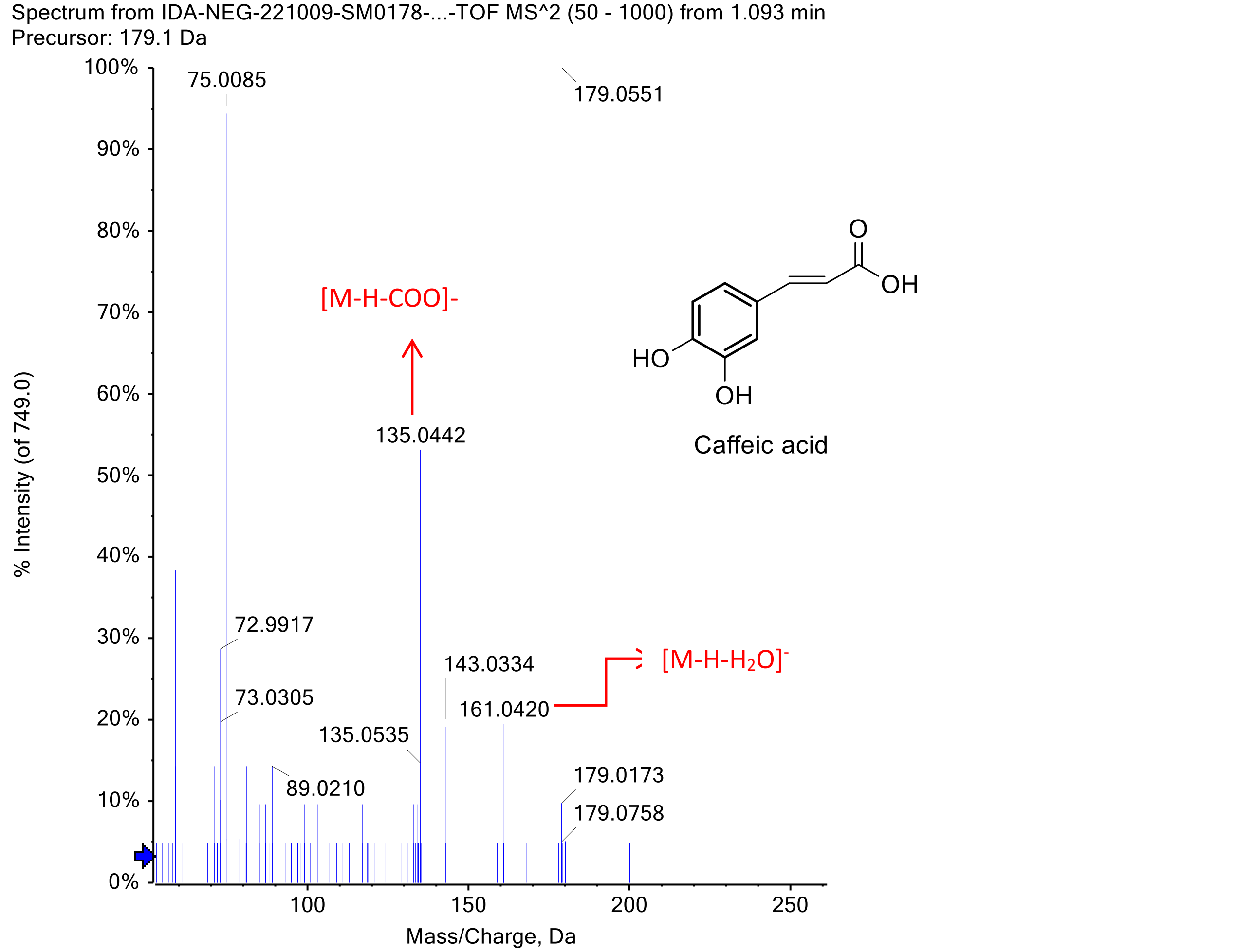

Figure S10. Caffeic acid –ve

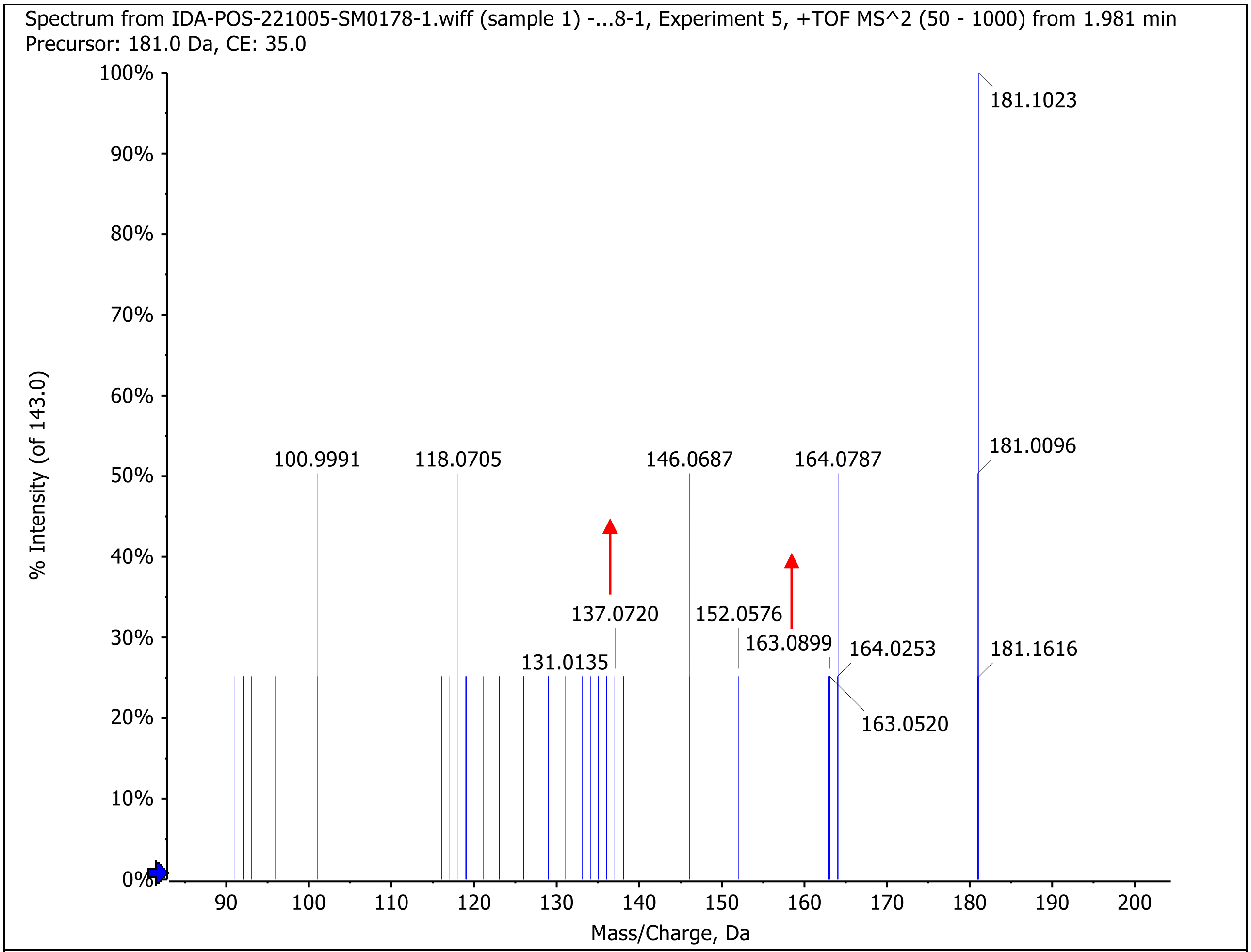

Figure S**11**. Caffeic acid +ve

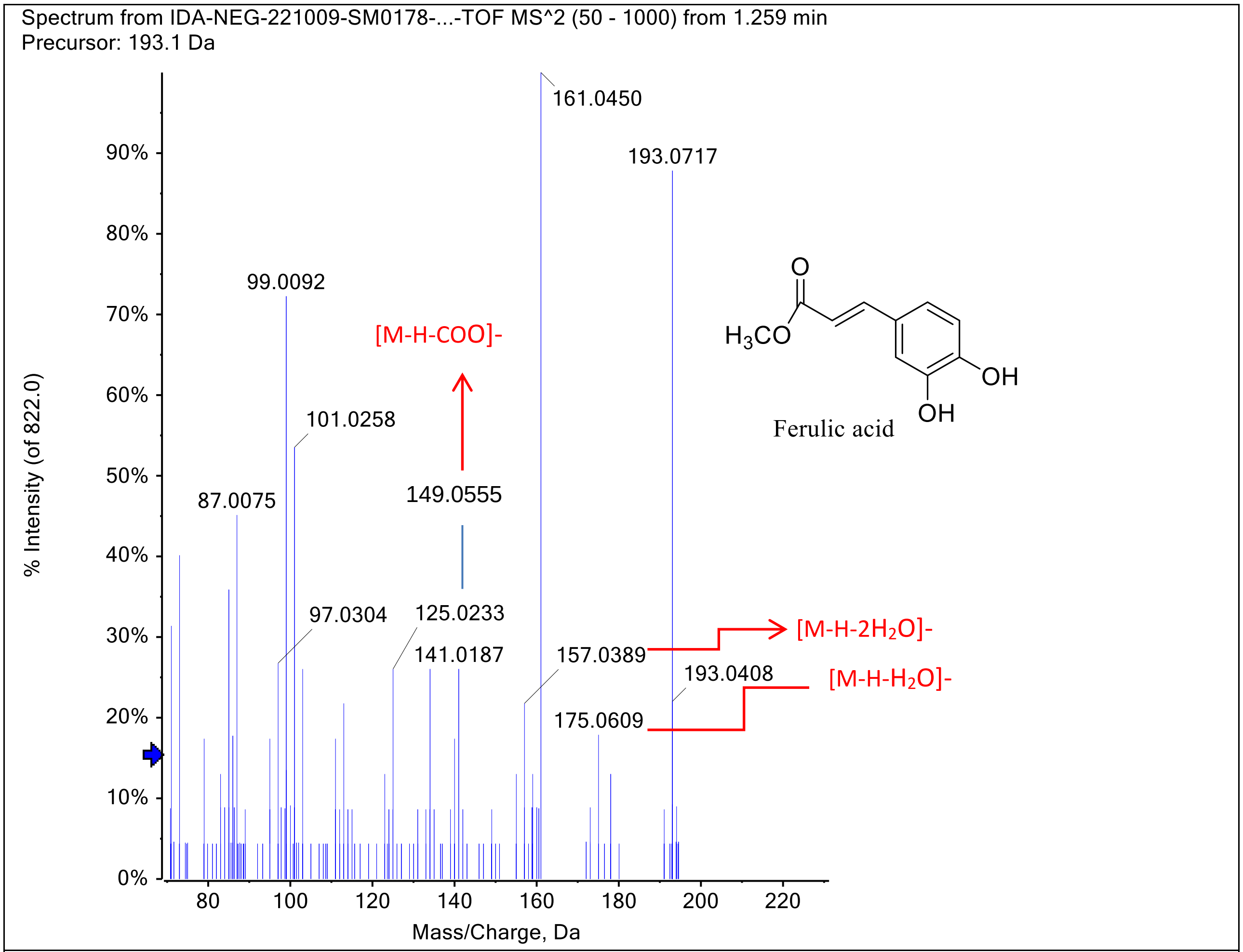

Figure S12. Ferulic acid(-ve)

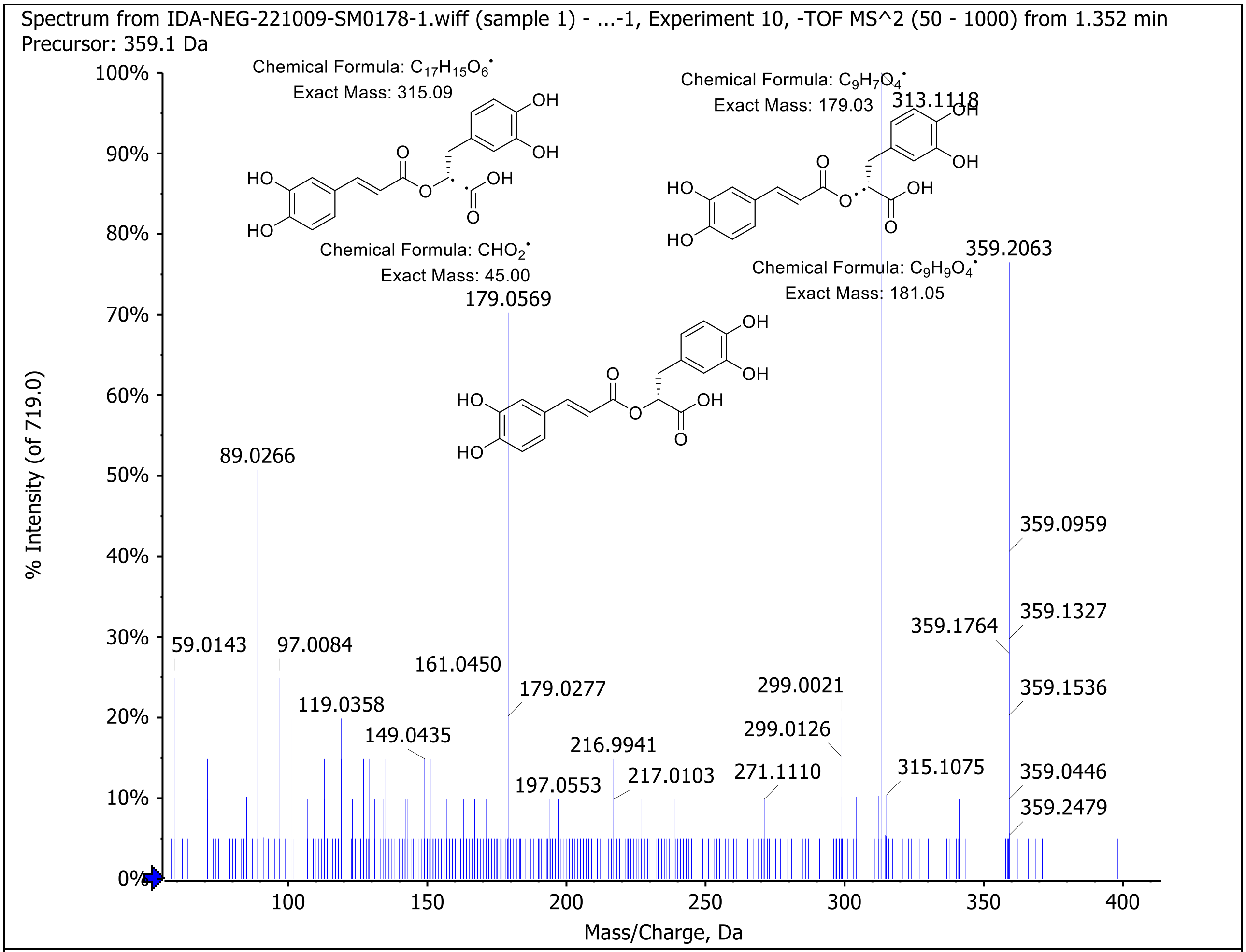

Figure S13. Rosmarinic acid (-ve)

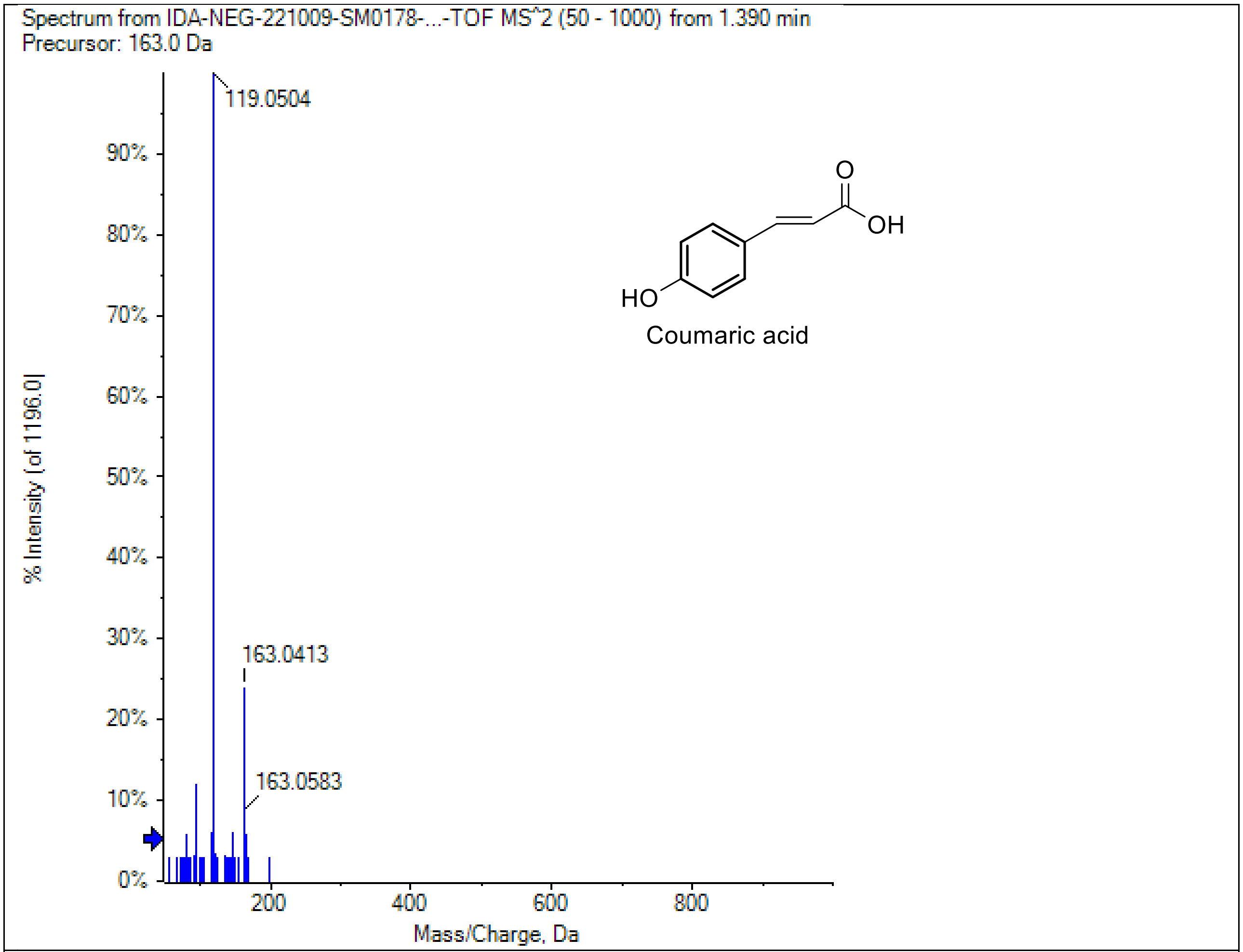

Figure S14. Coumaric acid –ve

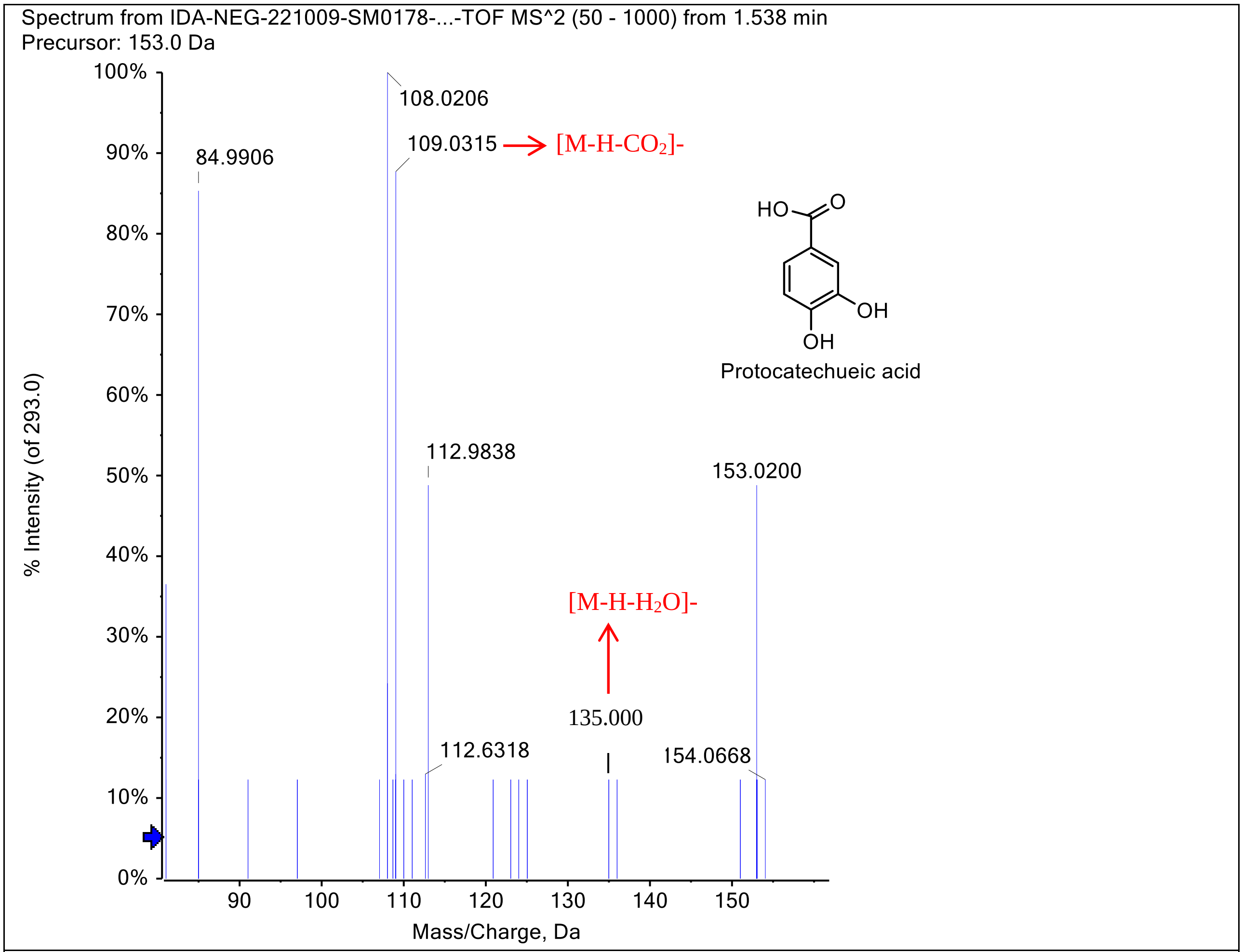

Figure S15. Protocatechueic acid –ve

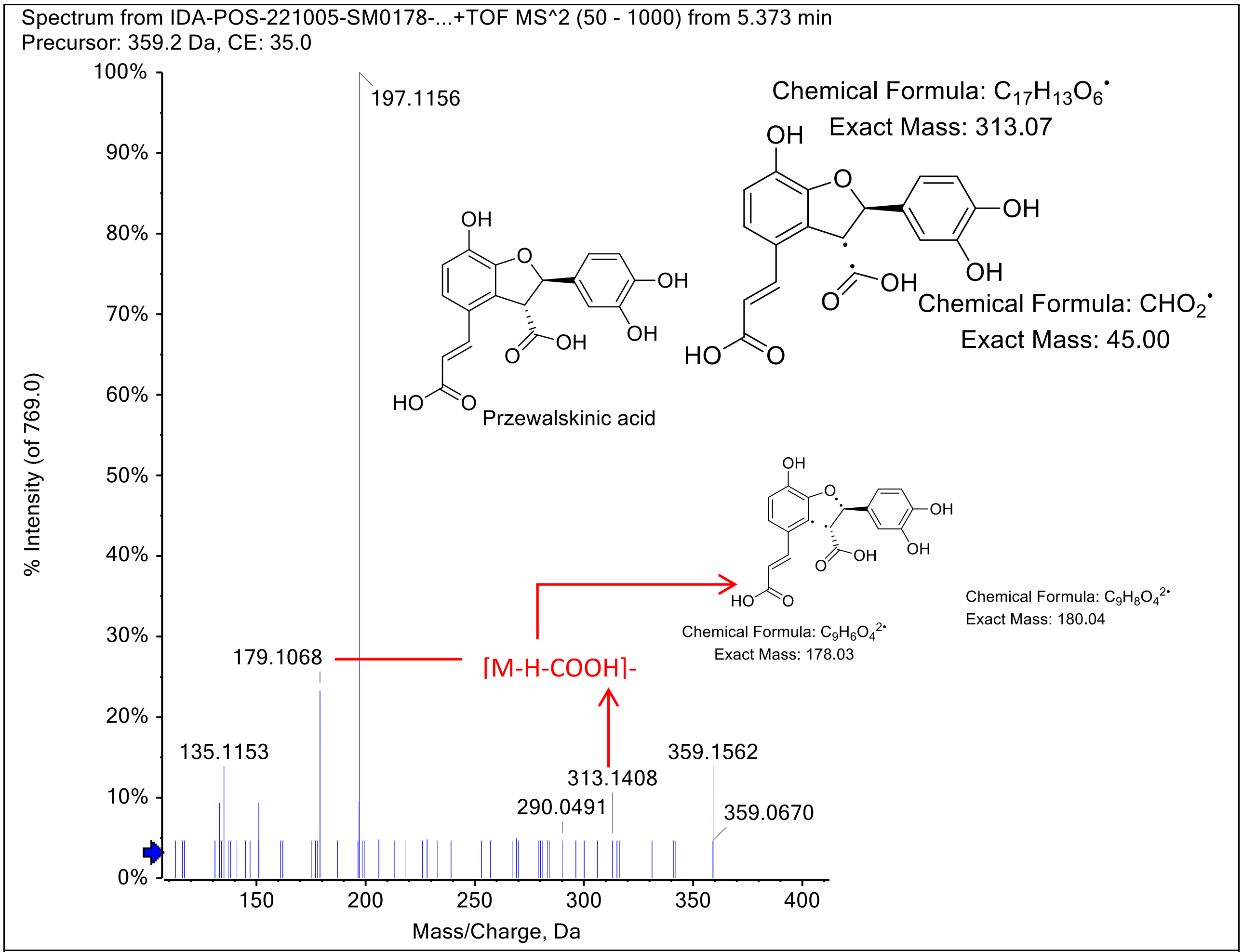

Figure S16. Przewalskinic acid (phenolic acid) +ve

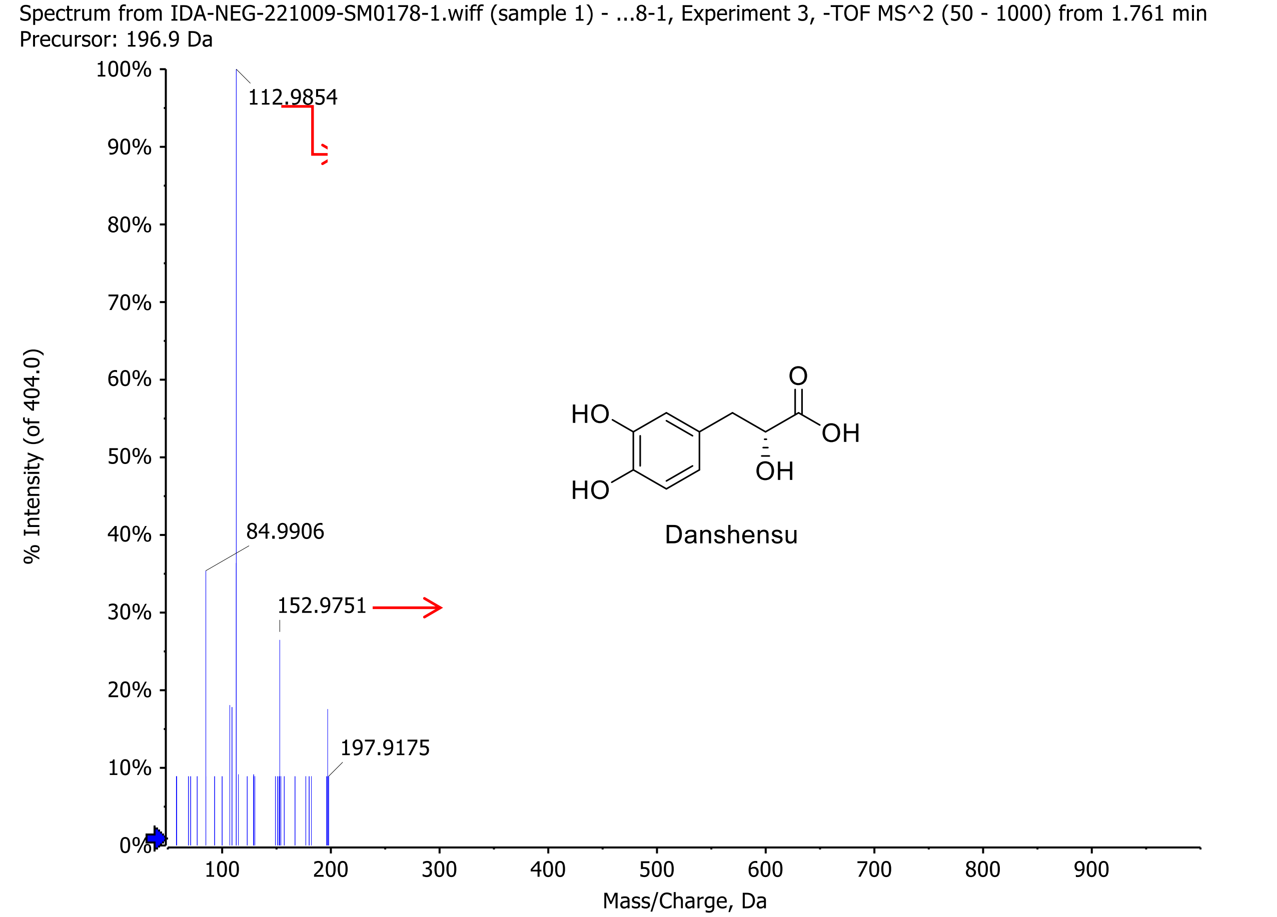

Figure S17. Danshensu (Hydroxycinnamic acid) –ve

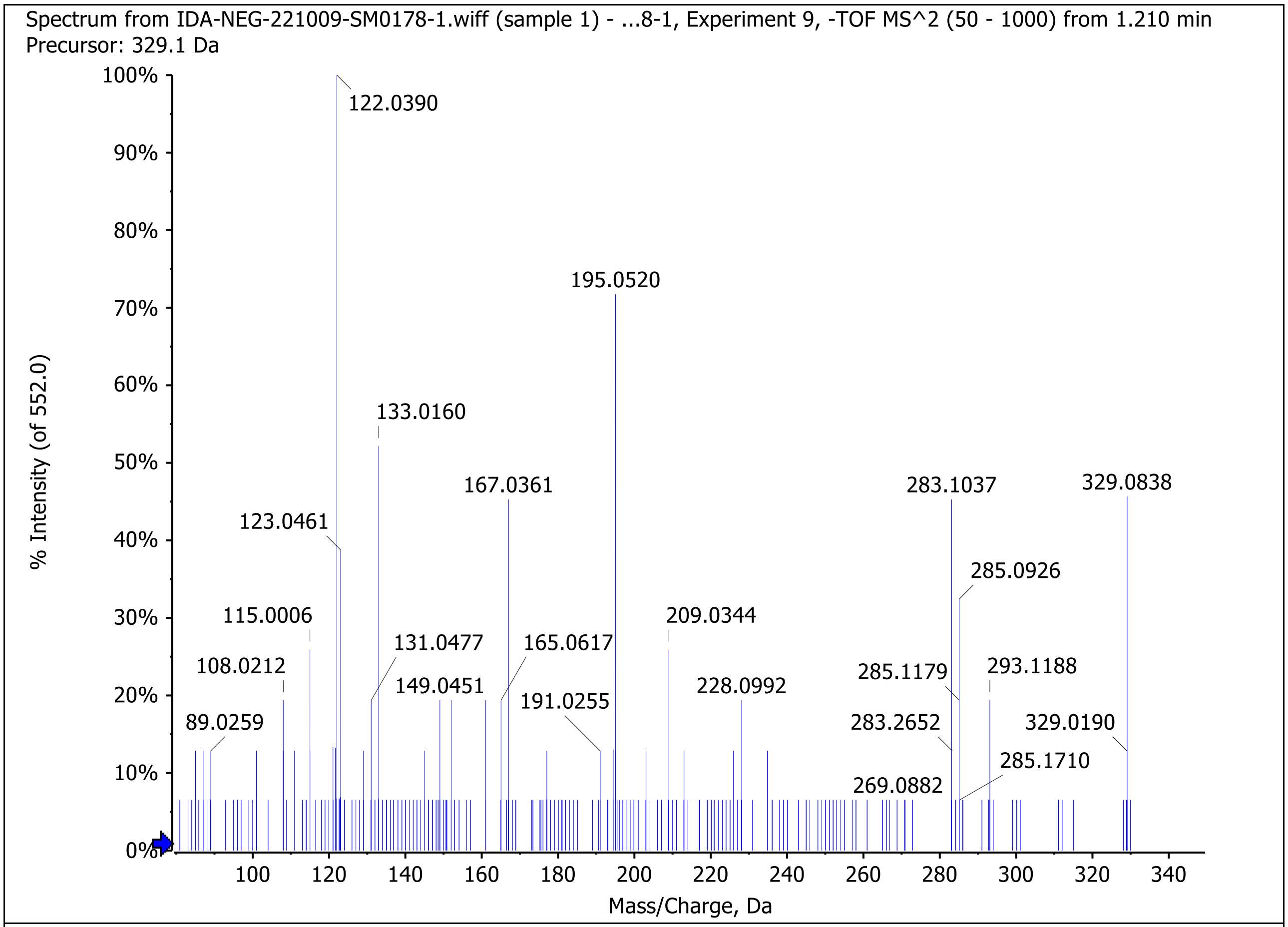

Figure S18. Vanillic acid-hexoside (-ve)

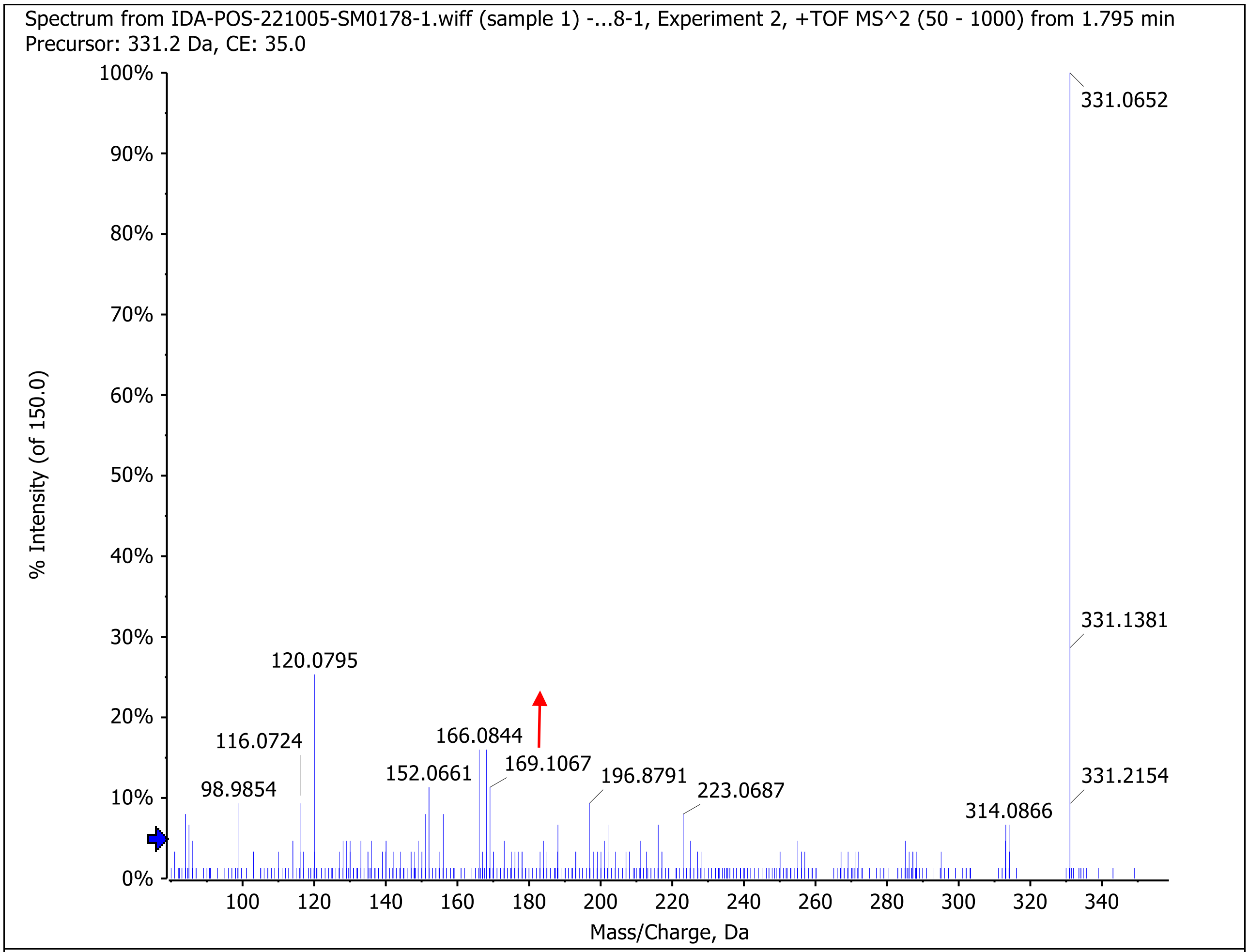

Figure S19. Vanillic acid-hexoside (-ve)

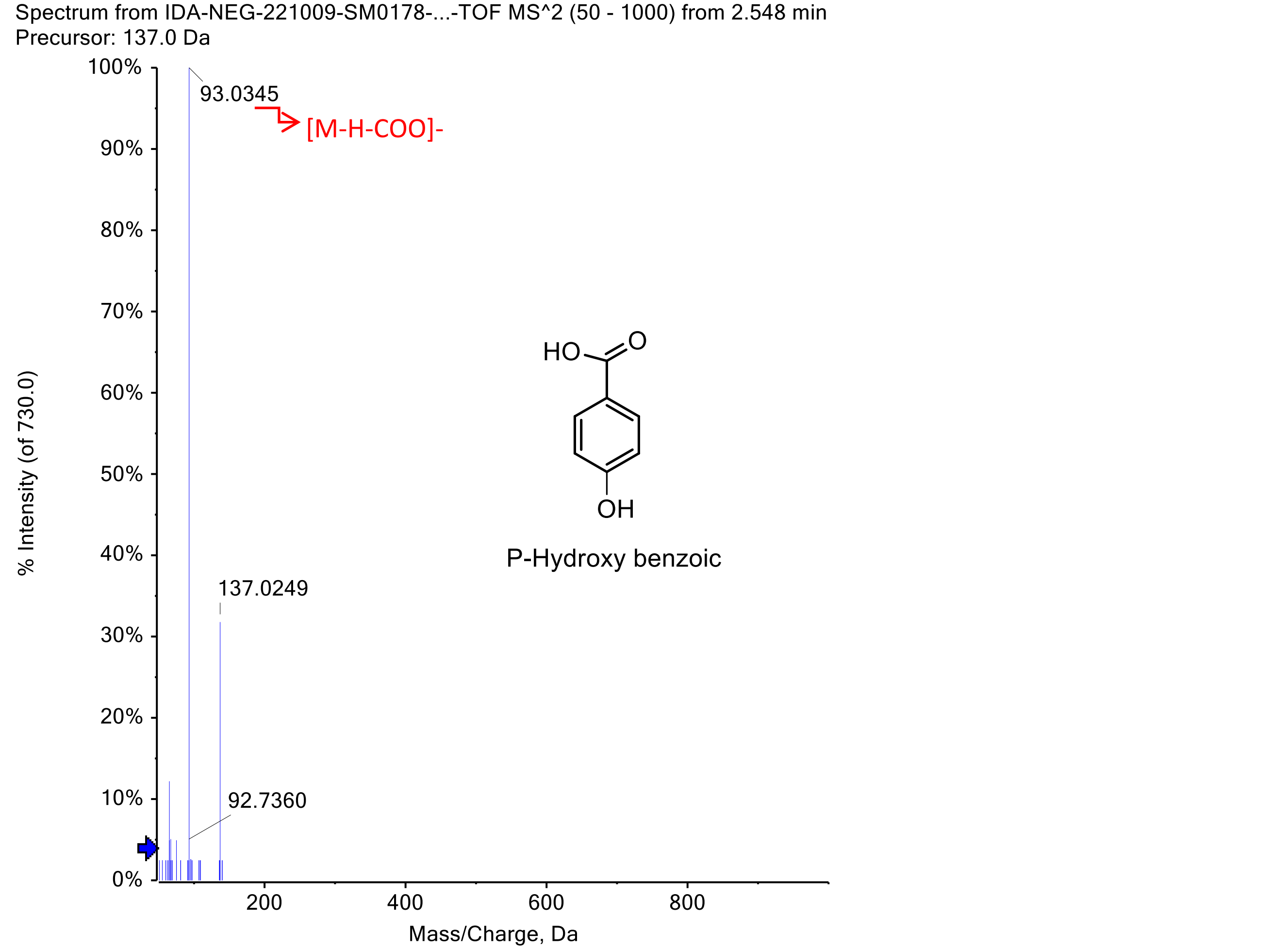

Figure S20. P-hydroxy benzoic acid

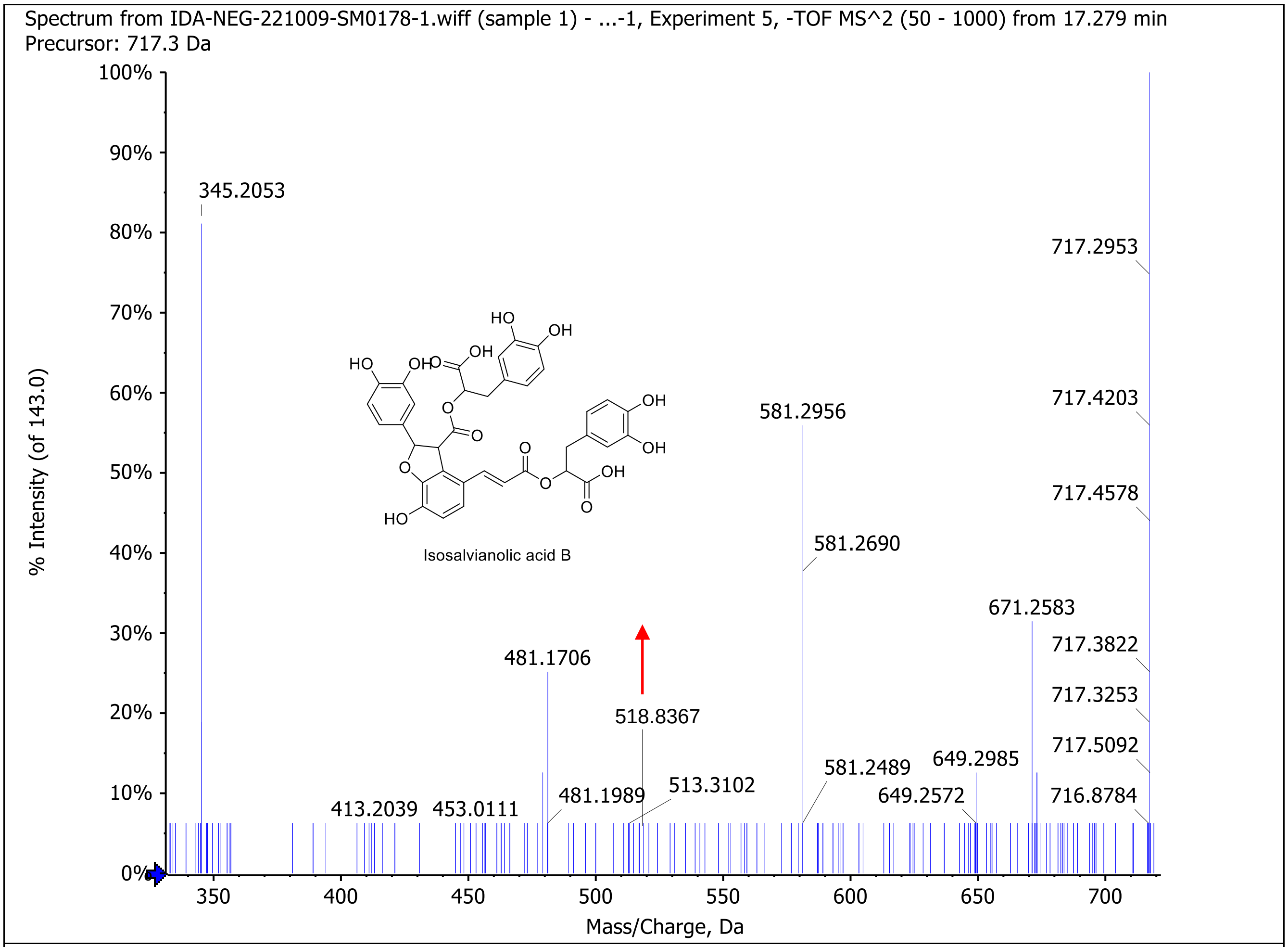

Figure S21. Isosalvianolic acid B

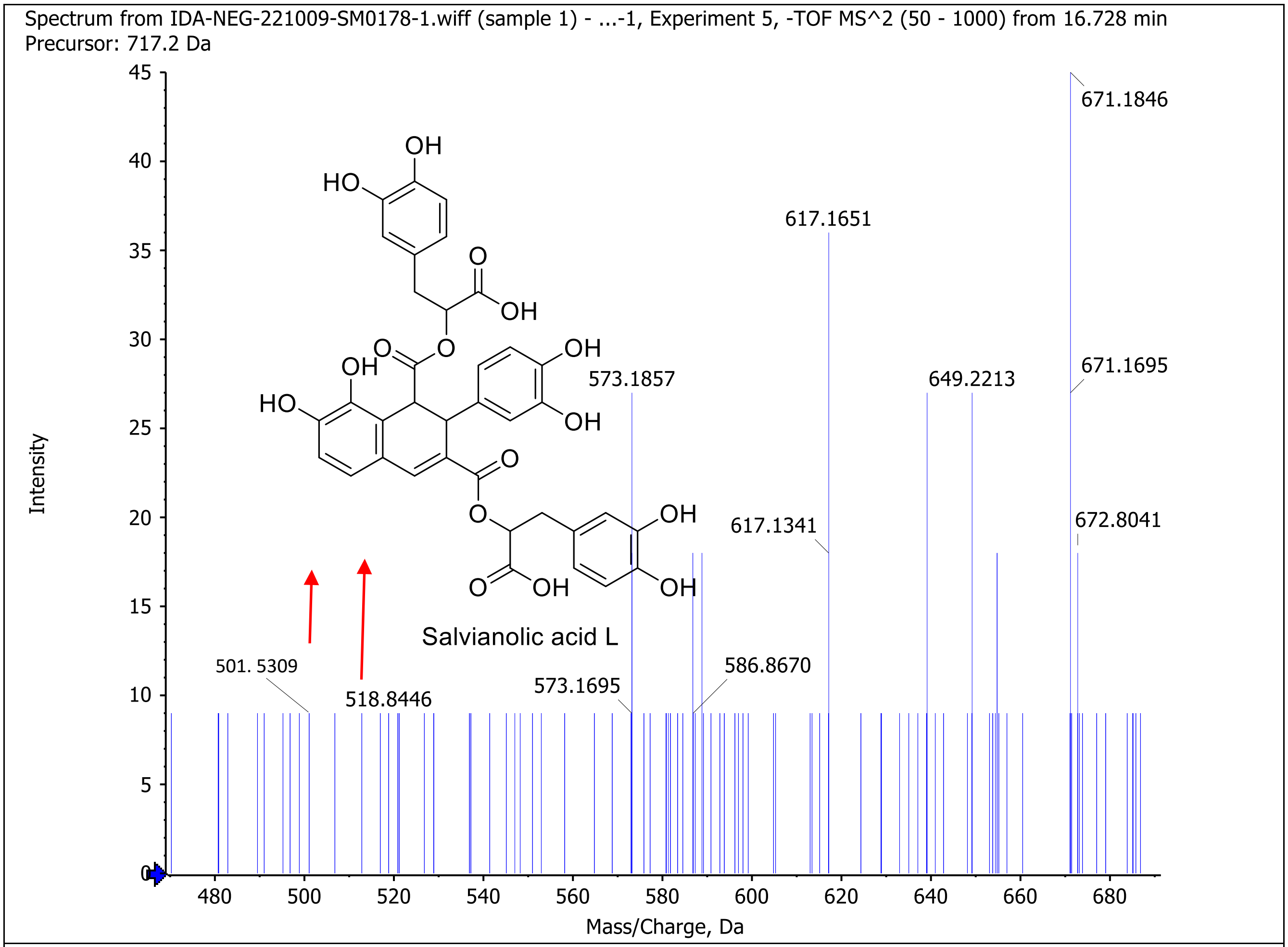

Figure S22. Salvianolic acid L
